# Species-wide distribution of highly polymorphic minisatellite markers suggests past and present genetic exchanges among house mouse subspecies

**DOI:** 10.1186/gb-2007-8-5-r80

**Published:** 2007-05-14

**Authors:** François Bonhomme, Eric Rivals, Annie Orth, Gemma R Grant, Alec J Jeffreys, Philippe RJ Bois

**Affiliations:** 1 Biologie Intégrative, ISEM CNRS Université de Montpellier 2 UMR 5554, Montpellier 34095, France; 2 LIRMM, CNRS Université de Montpellier 2 UMR 5506, rue Ada, Montpellier 34392 Cedex 5, France; 3 Department of Genetics, University of Leicester, Leicester LE1 7RH, UK; 4 The Scripps Research Institute, Department of Cancer Biology, Genome Plasticity Laboratory, Parkside Drive, Jupiter, Florida 33458, USA

## Abstract

**Background:**

Four hypervariable minisatellite loci were scored on a panel of 116 individuals of various geographical origins representing a large part of the diversity present in house mouse subspecies. Internal structures of alleles were determined by minisatellite variant repeat mapping PCR to produce maps of intermingled patterns of variant repeats along the repeat array. To reconstruct the genealogy of these arrays of variable length, the specifically designed software MS_Align was used to estimate molecular divergences, graphically represented as neighbor-joining trees.

**Results:**

Given the high haplotypic diversity detected (mean H_e_ = 0.962), these minisatellite trees proved to be highly informative for tracing past and present genetic exchanges. Examples of identical or nearly identical alleles were found across subspecies and in geographically very distant locations, together with poor lineage sorting among subspecies except for the X-chromosome locus MMS30 in Mus mus musculus. Given the high mutation rate of mouse minisatellite loci, this picture cannot be interpreted only with simple splitting events followed by retention of polymorphism, but implies recurrent gene flow between already differentiated entities.

**Conclusion:**

This strongly suggests that, at least for the chromosomal regions under scrutiny, wild house mouse subspecies constitute a set of interrelated gene pools still connected through long range gene flow or genetic exchanges occurring in the various contact zones existing nowadays or that have existed in the past. Identifying genomic regions that do not follow this pattern will be a challenging task for pinpointing genes important for speciation.

## Abstract 2

Global analysis of four minisatellite loci in House Mouse reveals unexpected long-range gene flow between populations and subspecies.

## Background

To address the significance of molecular polymorphisms, one option is to look at their distribution at population-, species-, and genus-wide scales. Polymorphic genetic features, such as variable number of tandem repeats (VNTRs) have long been considered to be the most informative markers due to their intrinsic high variability [1]. Minisatellites are particularly informative, as shown by their early use in forensics and paternity testing in humans [2]. Their very high level of variability made them ideal for DNA fingerprinting, linkage analysis, and population studies [3]. While semi-automated PCR analysis of microsatellites has now largely replaced minisatellite-based systems, DNA typing of minisatellites still provides a powerful and highly discriminating tool. Unlike microsatellites that are composed of short repeats of a few base pairs (typically 1 to 6 bp), minisatellites are intermingled arrays of usually GC-rich variant repeats ranging from 10 to over 100 bp depending on the locus, and with array lengths varying from 100 bp to over 20 kilobases (kb). Intermingled patterns of variant repeats along the array can be charted by minisatellite variant repeat mapping by PCR (MVR-PCR) to provide exquisitely detailed information on internal allele structure. This strategy has been used extensively at human hypervariable minisatellites, with germline mutation rates greater than 0.5% per gamete, to obtain crucial information needed to understand repeat turnover processes at these VNTRs (reviewed in [4,5]). Due to the unstable nature of minisatellites together with the frequently complex inter-allelic conversion-like germline mutation process, pedigree analysis can be performed for only a limited number of generations before it becomes impossible to trace back the original allele structure.

In the mouse genome, the situation appears to be more favorable for pedigree and genealogy analysis. Systematic isolation has identified human-like minisatellite loci (for example, GC-rich, highly polymorphic) [6]. However, none were found to be hypermutable. Analyses of mouse semen DNA demonstrated that mutant alleles were rare, with mutation frequencies at or below 5 × 10^-6 ^per sperm. However, these frequencies are an underestimate since mutations involving gain or loss of one to three repeats, likely to be the most common type of mutation, would have been lost during mutant enrichment by DNA fractionation [7]. Also, female mutation rates are not known. In contrast to human minisatellites, mouse sperm mutants arise by simple intra-allelic duplication and deletion, similar to those observed in human blood DNA [7,8]. This combination of high polymorphism, lower mutation rate, and relatively simple intra-allelic turnover mechanisms make mouse minisatellites potentially highly informative for species-wide population studies. Nevertheless, reconstructing the genealogy of alleles is hampered by the fact that aligning their sequences is difficult. Recently, however, development of new algorithms specifically designed to treat tandem repeat data has made analysis of large MVR datasets possible (MS_Align; [9]). This allows quantification of molecular divergence between alleles and renders these information-rich loci amenable to phylogenetic analysis. This allows the unique properties of rapid simple mutation and complex internal structure at minisatellites to be exploited to provide far more informative systems compared to classic markers such as non-repetitive DNA or microsatellites.

We therefore used MVR-PCR together with the MS_Align algorithm to study for the first time the distribution of allelic variants at four different minisatellite loci in the house mouse (*Mus musculus*). This species has radiated outside its original range within the last 0.5 million years, leaving at its periphery three well recognized subspecies with recent ancestry (*M. m. domesticus*, *M. m. musculus*, and *M. m. castaneus*) and populations of a more ancient descent at its center [10]. Its range has more recently expanded outside Eurasia because of commensalism with man [11], allowing many recent secondary contacts to occur, leading to a certain amount of re-admixture. The possibility of a gene re-entering a gene pool depends strongly on the kind of selective pressures exerted on it during its co-evolution from its original background. The occurrence of progressive incompatibilities building up during the course of allele divergence (so called Dobzhansky-Muller incompatibilities) may impede this phenomenon. At the opposite end of the spectrum, facilitation may occur if some strong selective advantage is provided by the gene irrespective of the recipient background. These contrasting possibilities will shape the coalescence of individual chromosomal segments when differentiated gene pools have co-existed for appreciable amounts of time, as in the house mouse. The question of allele circulation throughout the species range is presently an important focus for understanding the impact of selective forces that shape complex eukaryotic genomes. However, for a standard nuclear DNA sequence the intra-specific nucleotide divergence is generally small, resulting in very short and poorly informative coalescent branches within subspecies. To characterize allele circulation among house mouse subspecies, we report intra-specific coalescence analysis at four minisatellite loci, MMS24, 26, 80, and 30 [4], located on chromosomes 7 (22 cM), 9 (68 cM and 79 cM), and X (43 cM), respectively, on a panel of 116 individuals of various geographical origins.

## Results

### Array size and map structure

**Figure 1:**
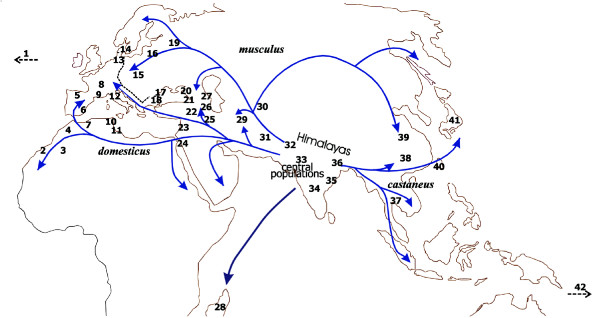
Geographical location of the localities sampled. 1, Lake Casitas, CA, USA; 2, Azzemour, Morocco; 3, Ouarzazate, Morocco; 4, Azrou, Morocco; 5, Leo'n prov., Spain; 6, Granada, Spain; 7, Oran, Algeria; 8, Ardèche, France; 9, Montpellier, France 10, Monastir, Bembla, M'saken, Tunisia; 11, Sfax, Tunisia; 12, Cascina Orcetto, Italy; 13, Ödis, Denmark; 14, Hov, Denmark; 15, Bohemia reg., Czech Republic; 16, Bialowieza, Poland; 17, Kranevo, Sokolovo, Bulgaria; 18, Vlas, Bulgaria; 19, Moscow, Russia; 20, Abkhasia prov., Georgia; 21, Adjaria prov., Georgia; 22, Van Lake, Turkey; 23, KefarGalim, Israel; 24, Cairo, Egypt; 25, Megri, Armenia; 26, Alazani, Chirackskaya, DidichChiraki, Gardabani, Lissi, Vachlavan, Tbilissi, Georgia; 27, Daghestan, Russia; 28, Antananarivo, Manakasina, Madagascar; 29, Mashhad, Kahkh, Birdjand, Iran; 30, Turkmenistan; 31, Gujarkhan, Islamabad, Tamapasabad, Rawalpindi, Pakistan; 32, Jalandhar, Bikaner, Delhi, India; 33, Pachmarhi, India; 34, Masinagudi, India; 35, Varanasi, India; 36, Gauhati, India; 37, PathumThani, Thailand; 38, Gansu prov., China; 39, Fuhai, China; 40, Taiwan; 41, Mishima, Japan; 42, Tahiti, French Polynesia.

**Figure 2:**
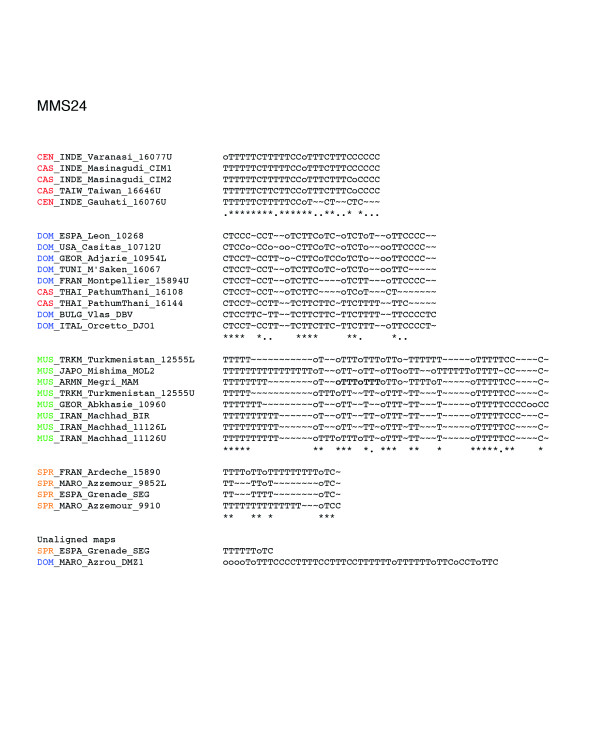
Maps of the internal structure of variant repeats for mouse minisatellite MMS24. Groups of similar haplotypes were chosen arbitrarily for the purpose of illustrating the maps' complexity. The groups correspond to clades in the trees of Figure 7. Their maps were aligned with the multiple alignment procedure MS_Alimul (E Rivals, unpublished) and the alignments edited manually. Under an alignment column, an asterisk indicates a complete conservation, while a period means that 60% of the variants in the column are identical. The alignments show which type of mutations occur between alleles, and where corresponding differences are located in the maps. For comparison, we also display for each locus one of the shortest and one of the longest or most complex alleles. Color code: *spretus*, orange; *domesticus*, blue; *castaneus*/cen, red; *musculus*, green.

**Figure 3:**
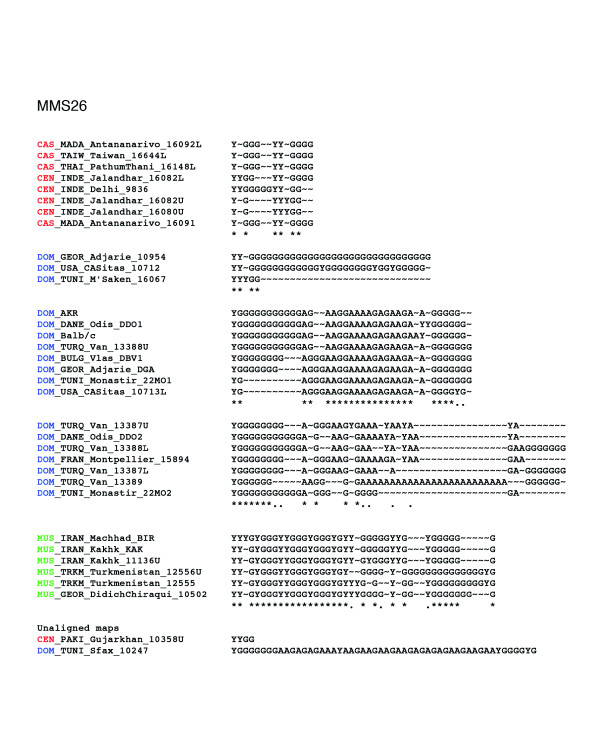
Maps of the internal structure of variant repeats for mouse minisatellite MMS26. Groups of similar haplotypes were chosen arbitrarily for the purpose of illustrating the maps' complexity. The groups correspond to clades in the trees of Figure 7. Their maps were aligned with the multiple alignment procedure MS_Alimul (E Rivals, unpublished) and the alignments edited manually. Under an alignment column, an asterisk indicates a complete conservation, while a period means that 60% of the variants in the column are identical. The alignments show which type of mutations occur between alleles, and where corresponding differences are located in the maps. For comparison, we also display for each locus one of the shortest and one of the longest or most complex alleles. Color code: *spretus*, orange; *domesticus*, blue; *castaneus*/cen, red; *musculus*, green.

**Figure 4:**
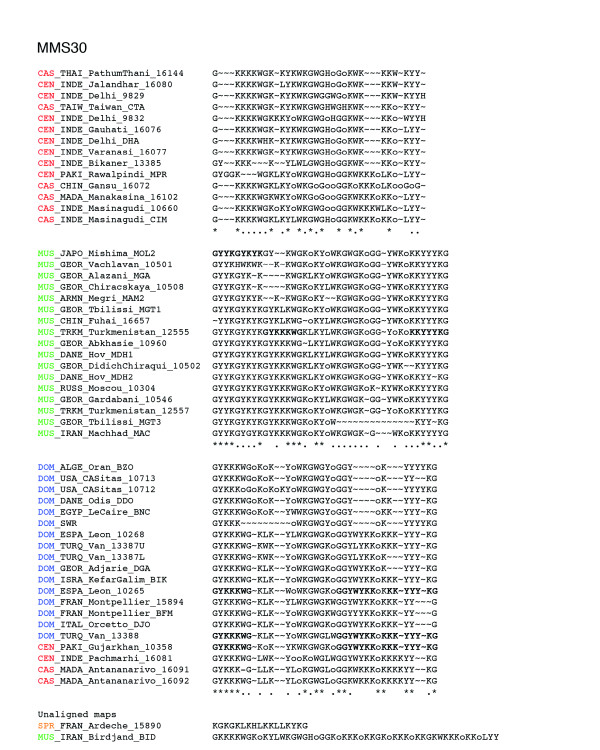
Maps of the internal structure of variant repeats for mouse minisatellite MMS30. For this locus, the alignments of *domesticus *haplotypes also comprise 4 CAS/CEN haplotypes. These *castaneus *and central haplotypes are clearly more similar to the *domesticus *alleles than to the group of CAS/CEN alleles in the top multiple alignment. The sequence motifs shared between these introgressed CAS/CEN haplotypes and the *domesticus *and/or the *musculus *haplotypes are shown in bold in a few maps. Groups of similar haplotypes were chosen arbitrarily for the purpose of illustrating the maps' complexity. The groups correspond to clades in the trees of Figure 7. Their maps were aligned with the multiple alignment procedure MS_Alimul (E Rivals, unpublished) and the alignments edited manually. Under an alignment column, an asterisk indicates a complete conservation, while a period means that 60% of the variants in the column are identical. The alignments show which type of mutations occur between alleles, and where corresponding differences are located in the maps. For comparison, we also display for each locus one of the shortest and one of the longest or most complex alleles. Color code: *spretus*, orange; *domesticus*, blue; *castaneus*/cen, red; *musculus*, green.

**Figure 5:**
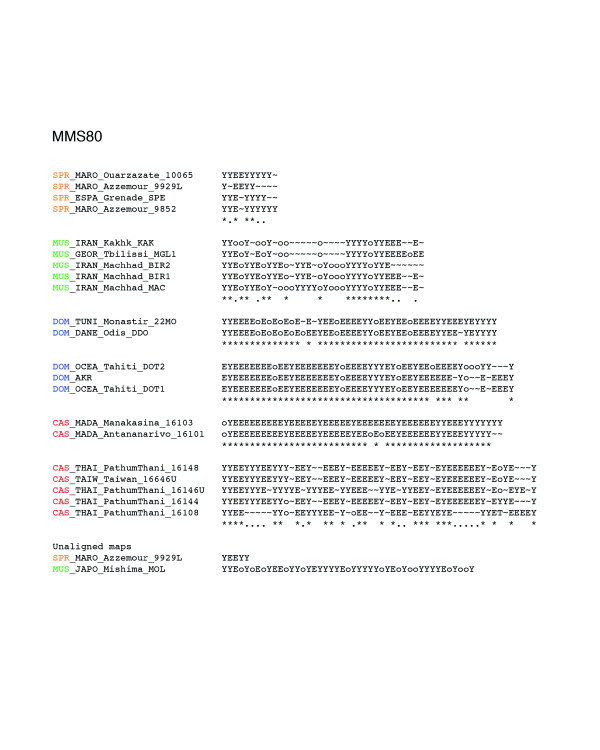
Maps of the internal structure of variant repeats for mouse minisatellite MMS80. Groups of similar haplotypes were chosen arbitrarily for the purpose of illustrating the maps' complexity. The groups correspond to clades in the trees of Figure 7. Their maps were aligned with the multiple alignment procedure MS_Alimul (E Rivals, unpublished) and the alignments edited manually. Under an alignment column, an asterisk indicates a complete conservation, while a period means that 60% of the variants in the column are identical. The alignments show which type of mutations occur between alleles, and where corresponding differences are located in the maps. For comparison, we also display for each locus one of the shortest and one of the longest or most complex alleles. Color code: *spretus*, orange; *domesticus*, blue; *castaneus*/cen, red; *musculus*, green.

The entire data set is available at [12]. The geographical origin of the mice used in this study is shown in Figure 1. The number of different alleles and overall allelic diversity is provided in Table 1 for each of the four minisatellite loci analyzed. All loci proved to be highly variable in length and array structure (He 0.90-0.99). Figures 2, 3, 4, 5 show examples of MVR structures encountered. DOM, MUS, CAS stand for *domesticus*, musculus and *castaneus *respectively, while CEN designates the less well defined central populations. For each locus, for the sake of graphical representation, we computed a multiple alignment according to the unpublished method of Rivals (MS_Alimul) of some representative MVR codes for each subspecies. While all haplotypes were employed in the pairwise estimation of genetic distance between haplotypes performed with MS_Align, computations with MS_Alimul were made for subsets of similar MVR maps, otherwise the proposed alignment would require too many gaps. We also included unaligned short and long alleles, as well as some of the more divergent alleles encountered. We supply for each locus the set of alleles whose MVR codes were identical as supplementary material at [12].

### Trees

**Figure 6:**
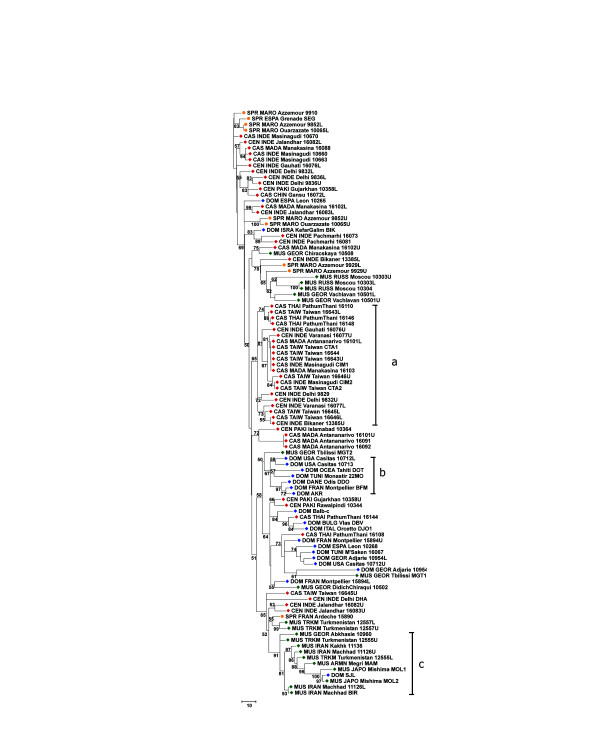
Most reliable coalescence obtained at locus MMS24. Neighbor-joining trees obtained from the matrices of allele alignment distances computed with the MS_Align pairwise alignment program [9]. For each internal edge, the corresponding confidence value Re (in the range [0,100]) is shown. The clades referred to by roman letters in parentheses in the text are indicated.

**Figure 7:**
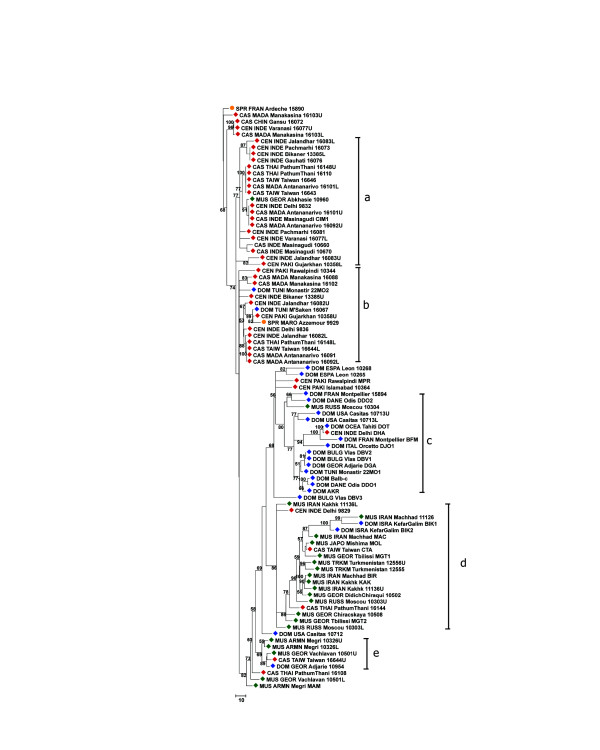
Most reliable coalescence obtained at locus MMS26. Neighbor-joining trees obtained from the matrices of allele alignment distances computed with the MS_Align pairwise alignment program [9]. For each internal edge, the corresponding confidence value Re (in the range [0,100]) is shown. The clades referred to by roman letters in parentheses in the text are indicated.

**Figure 8:**
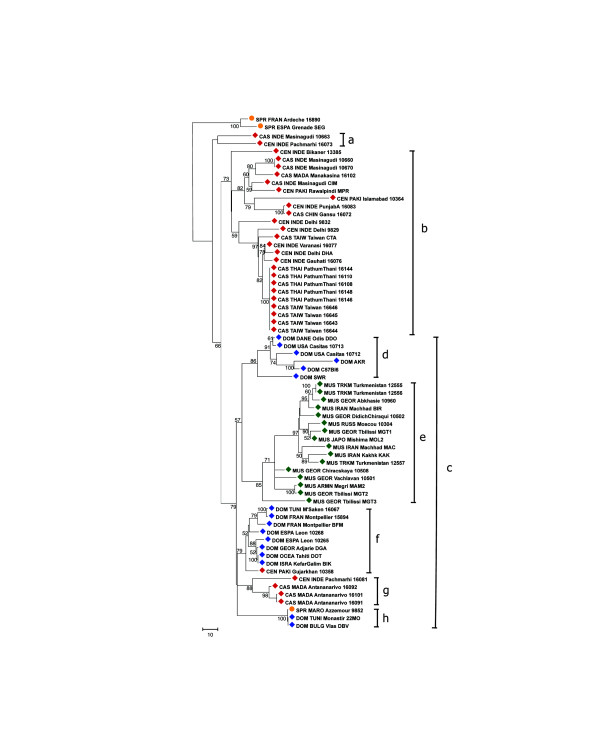
Most reliable coalescence obtained at locus MMS30. Neighbor-joining trees obtained from the matrices of allele alignment distances computed with the MS_Align pairwise alignment program [9]. For each internal edge, the corresponding confidence value Re (in the range [0,100]) is shown. The clades referred to by roman letters in parentheses in the text are indicated.

**Figure 9:**
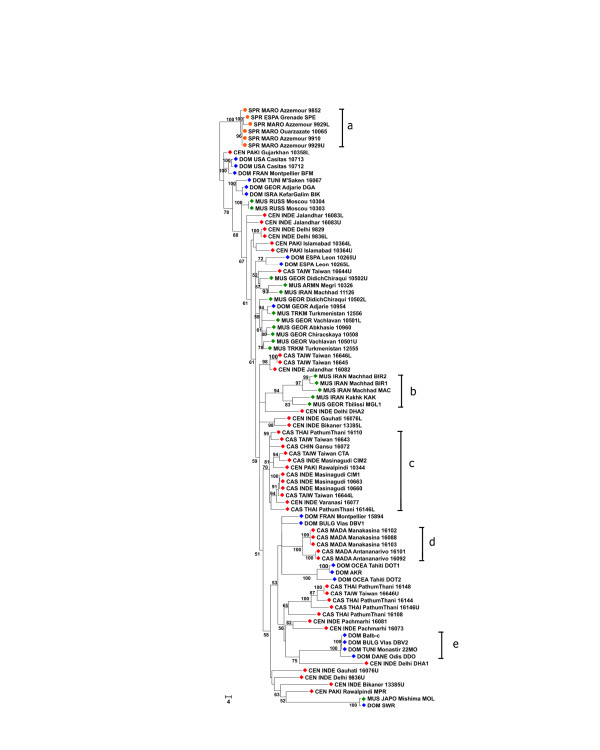
Most reliable coalescence obtained at locus MMS80. Neighbor-joining trees obtained from the matrices of allele alignment distances computed with the MS_Align pairwise alignment program [9]. For each internal edge, the corresponding confidence value Re (in the range [0,100]) is shown. The clades referred to by roman letters in parentheses in the text are indicated.

Figures 6, 7, 8, 9 show the coalescence patterns observed at each locus across a reduced panel of haplotypes. For the sake of legibility, only the locales analyzed for at least three loci have been included in the trees, but the results presented below were qualitatively identical to what could be inferred from the complete set of individuals. One striking feature is the variable degree of subspecific coalescence observed, which goes from almost complete resolution of the *domesticus*, *musculus*, and *castaneus *clades for sex chromosome locus MMS30 (Figure 8) to a much more interspersed situation for MMS24 (Figure 6). Nevertheless in all four trees, small clades of almost pure subspecific composition could be identified. These small clades were robust with respect to variations in penalty parameters used to align alleles (see Materials and methods); this robustness can be observed when comparing for each locus a sub-optimal tree (given in supplementary Figure S3 at [12]) and the corresponding optimal tree of Figures 6 to 9. Below, we list noticeable, well supported clades in each tree.

The MMS30 tree (Figure 8) offers the best subspecific resolution. When rooted by two European *spretus *alleles, starting from the top node we first observe a not very solidly placed subtree with two CAS/CEN alleles (a), and a reasonably well-supported clade (Re = 0.66; see Materials and methods for a description of Re) encompassing all the rest. This further splits into two equally well-supported clades (Re = 0.73 (b) and Re = 0.79 (c)). The uppermost one contains 24 out of 30 CAS/CEN alleles, while the bottommost constitutes a paraphyletic grouping of three independent DOM clades (with Re = 0.86 (d), 0.79 (f), and 1.00 (h)), a small CAS/CEN clade of four haplotypes (Re = 0.88 (g)), and a well defined MUS clade (Re = 0.85 (e)) branching out between two subtrees containing DOM haplotypes. The *musculus *subspecies is thus the only one to appear monophyletic. In the '(f) *domesticus *subtree, one also observes one CEN haplotype (CEN_PAKI_Gujarkhan_10358). The case of these CAS/CEN 'intruders' in the *domesticus *subtree will be discussed further below. Moreover, a *spretus *haplotype, SPR_MARO_Azzemour_9852, is clustered with two *domesticus *haplotypes in clade (h) since they share exactly the same MVR map. This haplotype differs completely from the other SPR alleles, and suggests interspecific hybridization as already demonstrated in this Moroccan locality [13].

The coalescent for locus MMS26 (Figure 7) displays a similar, but somewhat fuzzier, pattern. Indeed, one still observes a split between a CAS/CEN part and a DOM part in which a large well-supported predominantly MUS clade (Re = 0.86 (d)) containing 15 out of 19 *musculus *haplotypes branches out. In the DOM/MUS part there is also a 15 haplotype subtree (Re = 0.80 (c)) containing 14 out of 23 *domesticus *individuals. However, in the upper part of the tree there are two main CAS/CEN clades (Re = 0.77 (a) and 0.53 (b)) that encompass 34 out of 43 CAS/CEN haplotypes, but also one MUS, two DOM, and one SPR alleles. In the bottom part, a small subtree (Re = 0.69 (e)) mixes DOM, CAS, and MUS haplotypes.

In contrast, the coalescence trees for loci MMS24 and MMS80 (Figures 6 and 9) both display interspersion of small and subspecies specific clades. For MMS80, the largest well-supported clades are the perfectly supported (Re = 1.00) monophyletic clade of *M. spretus *(a) haplotypes, and the homogenous clade of 12 CAS/CEN haplotypes (Re = 0.70 (c)). Other instances of well-supported specific clades for MMS80 include: a subtree of five *musculus *haplotypes originating from Iran and Georgia (Re = 0.94 (b)), a clade of five *castaneus *haplotypes from Madagascar (Re = 1.00 (d)) and a clade of four *domesticus *haplotypes from Tunisia, Bulgaria, and Denmark (Re = 1.00 (e)). For MMS24, the pattern is similar, although some of the clades are somehow larger. Noticeable are (i), a homogenous clade of 21 CAS/CEN haplotypes (Re = 0.65 (a)), a homogenous clade of 7 *domesticus *haplotypes (Re = 0.67 (b)), and a clade of 10 *musculus *haplotypes with one laboratory strain *domesticus *allele (Re = 0.91 (c)). The remainder of the tree shows a high level of interspersion. Between clades (b) and (c), one notices a subtree containing mostly *domesticus *but also two *castaneus *alleles, CAS_THAI_Pathumtani_16108 and CAS_THAI_Pathumtani_16144. These 'intruders' exhibit a high level of similarity to *domesticus *alleles as testified by their average distances to the set of alleles of each *Mus musculus *subspecies: 40 to DOM and 52 to CAS for allele 16108, and 37 to DOM and 45 to CAS for allele 16144 (see supplementary Table S2 at [12]). Indeed, they are included in the multiple alignment of DOM alleles of Figure 2, where their similarity to *domesticus *alleles and their dissimilarity to other CAS/CEN haplotypes becomes apparent. Such intruders, which exist at all loci and cannot be interpreted as artifacts (since they are similar but nevertheless different from alleles of another subspecies), highlight the capacity of the alignment program to correctly handle complex cases. (Examples of intruders at all loci but MMS30 are listed in supplementary Table S2).

In all four trees, the nearest neighbors of *M. spretus *haplotypes are CAS/CEN haplotypes. Moreover, the MMS26 and MMS30 trees agree on the split CAS/CEN-SPR against DOM-MUS. It is interesting that MMS26, 30, and 80 have similar variance accounted for (VAF) values (0.92, 0.93, 0.91 respectively) but different patterns of subspecific coalescence.

### Introgressed CAS/CEN haplotypes at locus MMS30

We mentioned above five *castaneus *and central haplotypes that appear inside the *domesticus/musculus *subtree of the MMS30 coalescence (Figure 8). We sought to understand why these haplotypes are not located in the CAS/CEN part of the tree with all other CAS/CEN haplotypes, and whether this reflected homoplasy and the over-simplification of the evolutionary model used in the alignment algorithm, or instead truly reflects alleles identical by descent. When looking at the alignment in Figure 4 for locus MMS30, it is striking that these intruder haplotypes differ considerably from the typical CAS/CEN MVR codes, and resemble much more the DOM or MUS haplotypes. Indeed, they share several sequence motifs (all displayed in bold in Figure 4) either with the DOM codes ('G-G- [YK]-W- [YK]-K-K' just before the 3'-most 'o'-motif) or with both the DOM and MUS codes ('K-K-Y(2,3)-K-G' at the 3' end, or 'G-Y-K-K-K-W-G' at the 5' end of DOM and at about the tenth position in MUS codes), and none of these motifs occur in the other CAS/CEN haplotypes. This supports clearly the neighborhood of DOM and MUS in the tree, and gives evidence that these 'intruders' do actually carry DOM-like haplotypes. In addition, note that the nine-variant motif ('G-Y-Y-K-G-Y-K-Y-K') at the 5' end of MUS haplotypes is specific for this subspecies.

### Identical haplotypes shared among geographically or taxonomically distant samples

Several identical or quasi-identical alleles are shared by geographically distant locations (Table S2 at [12]). For instance, at locus MMS24, allele DOM_TUNI_Sfax_10247L (CTTCCCCCCCCTTCTTTCTTTTToTTCC) is identical to DOM_USA_Casitas_10712L, while DOM_FRAN_Montpellier_BFM (CTTCCCCCCCoTToTTTCTTTTTTTTCCT) differs from DOM_DANE_Odis_DDO (CTTCCCCCCCoTT***T***TTTCTTTTTTTTCCT) by a single mutation (in bold italics). More surprisingly, CAS_CHIN_Gansu_16072L (CTTTCTTC) is just one T shorter than DOM_MARO_Azrou_DMZ2 (CTTTCTTCT). Even more unexpectedly, DOM_BULG_Vlas_DBV, DOM_TUNI_Monastir_22MO, and SPR_MARO_Azzemour_9852 share the same haplotype (GYKKKGWGKoGGYWYKKoKKKYYYKG) at this locus of the X chromosome. There are many other examples where identical haplotypes are shared among geographically distant subspecies, as shown at tree tips or in the complete data set (Table S1 at [12]). Occasionally, some haplotypes may be over-represented and geographically widespread. A striking example is the MMS30 haplotype (GKKKKWGKKYKWKGWGHoGoKWKKKWKYY), which is encountered 28 times in Taiwan and Madagascar, or the MMS24 haplotype (TTTTTTCTTTTTCCoTTTCTTTCCCCCC), which is encountered 10 times in India, Taiwan, and Madagascar.

## Discussion

### Haplotype diversity and mutation rates

From the numbers of alleles and overall allelic diversities given in Table 1, the locus with the smallest diversity is the X-linked MMS30, which is consistent with the smaller effective size of the X-chromosome compared to autosomes (a theoretical three-quarter ratio). Taking this into account, the diversity values in Table 1 are remarkably similar at each locus, which may reflect a globally uniform mutation rate at mouse minisatellites. This is consistent with the fact that the optimal trees were obtained with similar mutation penalty parameters for all loci.

MMS80 is one of the most unstable loci. Its mutation frequency has been directly estimated in the wild derived strain DHA male germline at 5.10^-6 ^per sperm, while no mutations were detected in Balb/c sperm [7]. However, considering an average haplotypic diversity inside subspecies for the three autosomal loci of 0.83 (calculation not shown) one can get an approximation of the evolutionary mutation rate μ from the equilibrium relationship H_e _= 4N_e_μ/(1 + 4N_e_μ) of *circa *2.5 × 10^-5^/generation for an effective size of N_e _= 5.10^4^. This last value can itself be deduced from the inverse relationship adapted for haploid genomes N_e _= H_e_/μ(1-H_e_) using an average mitochondrial D-Loop nucleotide diversity inside subspecies of 0.5% (from [14]) and a D-Loop mutation rate of 10^-7^/nucleotides/generation (from [15]). If Ne = 5.10^4 ^is an overestimate in the mouse, then the MMS mutation rate may be even higher. One reason for this discrepancy with published data may reside in the method for isolating mutant alleles by size-enrichment small-pool PCR (SESP-PCR). This did not permit the detection of length variations smaller than two or three repeats, nor mutations that did not affect the size of the array [7]. Nevertheless, the mutation rate of the mouse minisatellite loci studied here is higher than previously reported by a factor of approximately 20.

### Homoplasy versus migration

In order to draw biological inference from the trees built from our MVR analysis, we have to address the issue of evolutionary noise due to the variable nature of the VNTRs used, specifically homoplasy arising by convergent evolution of allele structures. Thus, the validity of the emplacement of, say, a small CAS/CEN subtree inside a DOM clade has to be questioned as well as the reality of similar or identical alleles shared by very distinct geographic samples. Several arguments suggest low levels of homoplasy in our data set. First, there is a good tree arboricity as measured by the VAF, being 86% for the tree of MMS24 and above 90% for the three others, and many clades are well supported with an Re index above 0.8. Second, there is a paucity of long branches inside various well-identified clades. In such clades, homoplasy on complex structures is expected to yield spurious imperfect matches between convergent alleles that would translate into long branches. The latter is not observed in the examples provided above, where the structural complexity of long alleles minimizes the likelihood of convergent evolution. This may not be the case, however, for very small alleles like the *spretus *ones, which are located toward the root of the tree. However, for long haplotypes, which are predominant in our data set, homoplasy may be discarded as the primary source of lack of reciprocal monophyly.

### Incomplete lineage sorting

On purely theoretical grounds, such intermingling could be due to incomplete lineage sorting leading to the preservation of ancestral polymorphisms in the various subspecific groups. The question is, therefore, whether or not the time elapsed since the divergence of *domesticus *and *musculus*, for instance, would allow a complete sorting of gene lineages. An estimation of this can be inferred from the coalescence of mitochondrial genes as reported in [14]. In this report, the intra-subspecific coalescence depth (estimated as twice the average intra-subspecific pairwise nucleotide divergence) is smaller than the divergence time (as estimated by the net divergence between taxa). These two values are 0.98% and 3.55%, respectively, for the DOM/MUS comparison, which makes a ratio of 0.27, much smaller than 1. Therefore, there is a clear monophyly of each mitochondrial lineage with a complete mitochondrial lineage sorting for these two subspecies (this is less clear for the CAS/CEN mitochondrial haplotypes [16]). For nuclear genes, the coalescence should be larger than for mitochondria due to increased effective population size, while the divergence time should be the same for all neutral genes. So the ratio of coalescence over divergence is expected to be four-fold larger than for the mitochondrial data set. Extrapolating from the mitochondrial divergences computed by [14], this would give a ratio of 1.08 for the DOM/MUS comparison and 1.75 and 2.35 for the DOM/CAS and MUS/CAS comparisons, respectively. Thus, we are, in principle, at the limit where one could predict complete coalescence of nuclear genes to eventually occur for *domesticus *and *musculus*, while incomplete lineage sorting is expected to occur for haplotypes retained in the CAS (and even more so in the CEN) coalescent. Note that if the coalescence of the mitochondrial genome has been reduced by selection as suggested for species with large effective sizes [17], our mitochondrial value would be an underestimate, and thus incomplete lineage sorting is even more likely for nuclear genes unless they are also subjected to selection. This fits well with what we observe in the trees since monophyly is never attained except for *musculus *at MMS30 and probably corresponds to the fact that *musculus *and *domesticus *subspecies are likely to have experienced evolutionarily smaller effective sizes while migrating out of the Indian subcontinent cradle than the central populations that supposedly occupied their distribution range for a longer time [16,18].

However, even if several molecular lineages were to be kept segregating for a long time inside the subspecies, they would have acquired autapomorphic mutations that could allow them to be distinguished easily, and would not yield close molecular similarity, such as seen here for some mouse minisatellites. With the mutation rate estimated above, the probability of having two haplotypes remaining identical after 50,000 generations of divergence is less than 1%, while after 10,000 generations only, one expects, on average, 5 mutations per lineage. This necessarily means that exchanges of alleles have occurred by migration, and that those migrations are not restricted to within subspecies. On the other hand, it is also possible to find little subclades of closely related regionalized alleles that testify to local evolution of a probably former foreign migrant haplotype, as exemplified in the Results section. This is plausible, since if hybrid zone can trigger genetic exchanges now, they can have done so even more in the past, when taxa were less diverged than they are now. Indeed, before expansion with agriculture during the Neolithic, all subspecies were very likely restricted to a much smaller region going from the Near East and the Fertile Crescent to the southern slopes of the Himalayas, Elbourz, and Caucasus, and maybe around the Black and Caspian seas, but they were not elsewhere (this is well documented for *domesticus *westward bound in, for example, [19] and the literature cited therein, and the same should apply for *musculus *and *castaneus *northward and eastward). So indeed, these subspecies were all rather close to each other and ready to form local hybrid zones at each expansion/contraction cycle due to Pleistocene glaciations.

Altogether, the general lack of monophyly of the three main subspecific groups, together with the traces of recent and less recent migration events among them, is most likely due to the permeability of the various subspecies' genomes to foreign alleles, at least for the loci considered here. Note that this is less so for the X-linked locus MMS30, which has the best resolution of subspecies coalescences and shows a lesser amount of exchange between subspecies. This is expected since sex-chromosomes have been shown to accumulate interspecific incompatibility genes at a higher rate than autosomes for several reasons, such as smaller effective population size, less recombination, sexual selection, and arms races imposed by segregation distortion and genome imprinting. This is in agreement with the recently reported analysis of molecular diversity of six X-linked and seven autosomal loci [20]. Nevertheless, we show that for the locus considered and its surrounding, the X chromosome inside *domesticus *exists under at least three variant forms (see above) that are not necessarily closer to each other than they are to the *musculus *one; this may reflect past exchanges and introgression, even for the X chromosome.

These results may appear to conflic with previously published data concerning the identity and genetic borders of the various entities inside *M. musculus*. Most of the previous literature, however, concerns mitochondrial DNA, and it is true that at the global scale and at first glance, the distribution of matrilines fits rather well with taxonomy, and that the Latin trinomens seem to correspond to three well-defined entities (some authors have even considered them as full species [14]). Nevertheless, there are some exceptions to this, with evidence of mitochondrial DNA admixture even rather far from hybrid zones. Moreover, reciprocal monophyly is not completely granted either; the rooting of the so-called 'oriental' matrilines [16,21] that would characterize what we term here CEN (for central) is clearly a complex and poorly resolved matter. On the other hand, nuclear sequence data are rarely available with the same configuration (that is, more than ten individuals per subspecies). So most of the time there are only one or two sequences per taxon, which is not enough to detect the amounts of reticulation such as we have seen. At last, these possibilities of genome wide gene exchanges are reflected by a growing number of single nucleotide polymorphism (SNP) data that show that except for few specific regions [22,23], the three archetypal subspecific genomes are still largely compatible, as exemplified by the mosaic constitution of laboratory strains themselves [22-24]. Moreover, these SNP studies show that, when focusing for instance on the comparison between wild-derived strains of *musculus *and *domesticus*, three kinds of chromosomal segments can be sketched: some where the two subspecies are maximally divergent; some where intra- and intersubpecific levels of SNPs are comparable and rather high; and some with both low divergence and low polymorphism. Since we have no reason to consider that these mostly non-coding SNPs have highly variable mutation rates, the last two categories are good candidates for encompassing a continuum of situations, from retention of purely ancestral polymorphism to recent exchanges, with all possible intermediate situations.

### Divergence among subspecies

If one computes net average divergence among groups estimated by the mutational steps measured along the tree (tree distance; not shown), one obtains the same picture at all loci, with CAS being closest to CEN (5.6 steps on average over all 4 loci), while DOM and MUS are always invariably closer to CEN than to CAS (alignment scores of 18.4 and 24.7 for DOM, and 20.8 and 26.5 for MUS), the divergence between them being 21.1. If one then considers the intra-group average divergence, the CAS haplotypes show a tendency to be less diversified (34.9 steps) than MUS (43.4), CEN (49.5), or DOM (54.2). The *castaneus *alleles thus show the least diversity while the *domesticus *ones show the most. This is in good concordance with the fact that *castaneus *is a peripheral subspecies most recently derived from the central populations. The fact that *domesticus *displays greater diversity than the central populations is somewhat surprising since the latter populations are thought to have a longer history in the same place than *domesticus*, *castaneus *or *musculus*. This may be due to the fact that DOM MMS alleles tend to be longer than the CEN alleles (30.5 repeats on average versus only 24.15 for CEN), which will inflate the intra-group divergence even if the coalescence time is indeed less.

## Conclusion

Our murine VNTR study shows that these complex DNA structures when handled in a meaningful way with adequate alignment tools can reveal informative evolutionary data on species-wide genetic flow. This may not be the case when using simple non-repetitious sequences that may not possess enough intrinsic variation as well as simpler tandem repeats like microsatellites where mutation history is readily lost through homoplasy. Therefore, minisatellite VNTRs comprise an invaluable tool to identify past and present exchanges within the species range. Indeed, we show using our panel of wild-caught and wild-derived mice that such is the case: generalized non-reciprocal monophyly as well as current and less recent secondary exchanges between subspecies are a reality. Interestingly, this is true even for the X-chromosome despite its demonstrated tendency to diverge faster than autosomes. Indeed, sex-chromosomes are, by nature, prone to accumulate incompatibility genes, which supposedly render it less likely to cross subspecies borders [25]. Our analysis reveals a complex history at the MMS30 X-linked locus, with multiple origins for the *castaneus *and *domesticus *haplotypes (Figure 8). Thus, these wild mice genomes constitute a set of interrelated gene pools that are still able to exchange genes from time to time, at least for the four chromosomal locations and the sample of wild genomes analyzed. Recent SNP studies also illustrate this point [22,23], although SNPs do not allow as refined an analysis of the coalescence of a particular point in the genome as MMS do, this last technique potentially constituting an interesting means of revealing past and present forces having shaped the distribution of their flanking region species-wide. The overall picture fits well with the supposed phylogeographic scenario in which *castaneus *is a recent offshoot from the so-called central populations that would have occupied the species' ancestral range, while *domesticus *and *musculus *would have diverged earlier when migrating out.

## Materials and methods

### Animals and DNAs

We selected 116 samples from the Montpellier DNA collection to represent the main subspecies of *M. musculus *and its central populations. Some individuals were not original wild-caught mice, but were the offspring of mice that were bred in closed colonies of a single origin in our genetic repository in Montpellier. Their geographical origin is shown on the map in Figure 1 and incorporated in the individual designation of haplotypes available in the first column of Table S1 in [12]. The number of individuals studied at each location may vary slightly from one locus to another. Several individuals of the closely related species *M. spretus *were taken as an outgroup. Altogether, 92, 90, 82, and 87 wild or wild-derived mice were scored for MMS24, 26, 30, and 80, respectively. Additionally, laboratory strains' DNA (AKR, C57Bl6, DBA/2, C3H, Balb/c, SWR and SJL) was used as standards and included in the study. Routine laboratory strategies were taken to reduce to a minimum any possibility of DNA contamination or mix ups with such small batch processing during DNA extraction.

### Molecular methods

MVR-PCR uses variant repeats within minisatellite loci to generate internal maps of minisatellite alleles by a simple PCR assay [26]. Prior to MVR mapping, mouse minisatellite alleles were amplified to visible level using specific flanking primers. Flanking primers were (nomenclature: -, 5' of the array; +, 3' of the array; distance in kb from the repeated array; F, forward; R, reverse): MMS30-0.02/F, 5'-CTGGGATAGATTCATGCACAGC-3'; MMS30+0.03/R, 5'-CCTGCCACATGGTTAGTTACCT-3'. Amplifying primers and PCR conditions for MMS24, 26 and 80 have been previously described [6]. MMS30 amplifications were carried out at 96°C for 30 s, 66°C for 30 s, 70°C for 3 minutes for 28 cycles as described elsewhere [3]. PCR products were resolved by electrophoresis through 0.8% agarose gels. Two- and three-state MVR-PCR were developed at four independent mouse minisatellite loci using the same methodology as previously established [6,26]. MMS30 MVR-PCR reactions were carried out at 96°C for 50 s, 56°C for 45 s, 70° for 3 minutes for 24 cycles. The 5' flanking primer was MMS30-0.02/F. Two-state MVR specific 3' primers together with their final concentration per reaction were MMS30/TAG-CT (Y repeat: 5'-tcatgcgtccatggtccggaATCTTCTGTATAGTGTGAA**CT**-3', 1 nM); MMS30/TAG-GT (K repeat: 5'-tcatgcgtccatggtccggaATCTTCTGTATAGTGTGAA**GT**-3', 1 nM); MMS30/TAG-GG (G repeat: 5'-tcatgcgtccatggtccggaATCTTCTGTATAGTGTGAA**GG**-3', 1 nM). Nucleotide variations between primers are highlighted in bold and the TAG primer sequence is in lower case. The TAG primer used was as previously described [26]. MVR-PCR conditions for MMS24, 26 and 80 can be found elsewhere [6]. All subsequent MVR-PCR manipulations, including two-state and three-state MVR mapping, gel electrophoresis and detection were carried out as previously described [6,7]. Again, particular care was taken to avoid mixing up of the various samples. Each MMS locus was processed in two phases and some DNA was typed at least twice. No discrepancy was observed.

### Description of the loci

We selected three autosomal (MMS24, 26 and 80) and one X-linked (MMS30) minisatellite (Table 2). MMS26 and 80 are both located in the subtelomeric region of chromosome 9, 4 Mb apart. All autosomal minisatellites were located in intronic regions (Table 2). While distal from the promoter region, it is possible that these intronic minisatellites may contain enhancer regions that could potentially affect gene expression. However, the wide range of size observed at these minisatellites in *M. musculus *would suggest only a minimal, if any, effect of these VNTRs on gene expression.

### Alignment of MVR maps, distance, and penalties

To recover the relationships between alleles observed at a locus, one needs to quantify the molecular divergence between their MVR maps. For each locus separately, we considered the set of MVR maps of all haplotypes represented in our sample. The alphabet of possible variants is defined by the MVR-PCR experiments, such that MVR maps are sequences written in locus specific alphabets. Simply counting the difference of length between alleles yields a poor estimate of allele divergence, as illustrated in Figures 2, 3, 4, 5 (two very different haplotypes may have the same length). Obviously, one needs to consider not only the number of variants, but also the sequence of variants. Classic alignment methods suitable for DNA sequences cannot be applied to MVR maps. Indeed, these methods count only point mutations and disregard the main source of sequence divergence in VNTR, namely the tandem duplication or contraction events. The tandem duplication of a variant copies a variant and inserts the copy next to the template (for example, G→GG), and the reciprocal event, the tandem contraction of a variant, deletes one among two identical adjacent variants (for example, GG→G). In VNTR evolution, tandem duplication and contraction are considered to be much more frequent than point mutations. Therefore, to compare MVR maps we use the alignment program MS_Align [9], whose mutational model comprises, beyond insertion, deletion, and substitution, also tandem duplication and tandem contraction of a variant. The difference between tandem duplication and insertion is that, in an insertion, the inserted variant is not required to be identical to its adjacent variants. The propensity of the different mutations in the output alignments is controlled by the parameter penalties assigned to each mutation by the user. In the scoring scheme, the penalties are denoted by M for a substitution, I for an insertion, D for a deletion, A for a tandem duplication, and C for a tandem contraction. Each penalty is independent of the variant involved in the mutation event and our model is symmetrical, that is, I = D and A = C. Note that once being introduced by a duplication, a variant may later be changed into another by a substitution (which altogether is like an insertion); thus, duplication followed by a substitution may be preferred to an insertion, depending on the penalties.

Now, given a scoring scheme that associates a penalty to each type of mutation event, MS_Align computes an optimal alignment of minimum score between two MVR maps. An optimal alignment is the sequence of mutations that transforms one map into the other and whose sum of penalties is minimum. The penalties sum of an alignment is called the alignment score or the distance (since it is a metric distance in the mathematical sense). We compared in a pairwise fashion all MVR maps of a set with MS_Align [9], and this yields a pairwise distance matrix (with one distance per allele pair).

A present limitation of this approach is the undifferentiated treatment of the null variant (a repeat unit that does not prime during PCR due to the presence of an unknown sequence variant), with all nulls being treated as identical. Another limitation is the restriction of duplications and contractions to operate on single variant, and not on blocks of consecutive variants; that is, if the evolution of one allele has involved a duplication of a block of several variants (for example, TCoT → TCoTCoT), then MS_Align will find the best optimal alignment with single variant duplications, but without block duplication, and will thus overestimate the alignment score. Some alleles do indeed show evidence of these larger-scale duplications, such as the reduplicated oTTT motif in the MMS24 allele MUS_ARMN_Megri_MAM from positions 11 to 18 (see the third multiple alignment for MMS24 in Figure 2).

### Inference of locus coalescence

For a given locus, these comparisons yield a distance matrix giving the alignment score between any pair of MVR maps. The alignment score is a distance metric in the mathematical sense. We use the distance matrix to reconstruct an evolutionary tree for the MVR maps using an improved neighbor-joining algorithm called FastME [27]. To determine the robustness of the obtained coalescence with respect to alignment parameters, we iterated this procedure for 40 different combinations of penalties: A = C = 1, M = 4, 5, 6, 8, 10, 12, 14, or 16, and I = D = 8, 10, 15, 20, or 25. The most influential criterion is the ratio between the amplification and substitution penalties; thus, we set arbitrarily A = C = 1 and let M vary. For VNTR loci, amplification or contraction of a single variant are the most frequent events and are more probable than a variant substitution; this is the rationale for the chosen penalties. As bootstrapping is not meaningful for this type of data, we use an alternative to assess the confidence of each tree and of each node in the tree. We computed two mathematical criteria [28], the VAF and the rate of elementary well-designed quartets (Re). The VAF quantifies the adequacy of representing the distances between maps by a tree; the Re of an internal edge measures the average level of confidence over all possible quadruplets of taxa linked by this edge. The tree Re is an average of the edges' Re over all internal edges; it gives a global confidence value for the whole tree. This enables us to select the most reliable trees and to see whether sub-optimal trees differ greatly from the optimal (robustness). The optimal trees were obtained with penalties M = 6, I = 20 for MMS24, M = 8, I = 20 for MMS26, M = 6, I = 20 for MMS30, and M = 6, I = 8 for MMS80. Re is the value reported on the trees. For each locus, the values of criteria of each parameter combination are reported in Table S4 of the supplementary material [12].
